# Association between cyclin-dependent kinase inhibitor 2B antisense RNA 1 and zinc finger homeobox 3 gene polymorphisms and COVID-19 severity

**DOI:** 10.1186/s12879-023-08564-7

**Published:** 2023-08-31

**Authors:** Eman A. Badr, Nesreen G. Elhelbawy, Alaa Osama Nagy, Amany A. Sultan, Shereen S. Elnaidany

**Affiliations:** 1 Medical Biochemistry & Molecular Biology, Menofia University, Shebin- El-Kom, Egypt; 2 Anaesthesiology, Intensive Care & Pain Management Departments, Faculty of Medicine, Menofia University, Shebin- El-Kom, Egypt

**Keywords:** COVID-19, CDKN2B-AS1, ZFHX3

## Abstract

**Background:**

There is no doubt about the cardiovascular complications of coronavirus disease 2019 (COVID-19). Several genetic studies have demonstrated an association between genetic variants in a region on chromosome 9p21 and in a region on chromosome 16q22 with myocardial infarction (MI) and atrial fibrillation (AF) accompanied by cerebral infarction (CI), respectively.

**Objectives:**

MI and CI susceptibility in patients with CDKN2B-AS1 and ZFHX3 polymorphisms, respectively, may have an effect on COVID-19 severity. We aimed to investigate whether there is an association between the cyclin-dependent kinase inhibitor 2B antisense RNA 1 (CDKN2B-AS1) rs1333049 and zinc finger homeobox 3 (ZFHX3) rs2106261 single nucleotide polymorphisms (SNPs) and the degree of COVID-19 severity.

**Subjects and methods:**

This current work was carried out on 360 subjects. They were classified into three groups: 90 **severe COVID-19 cases**, 90 **moderate COVID-19 cases** and 180 age- and gender-matched **healthy controls**. All subjects underwent genotyping of CDKN2B-AS1 (rs1333049) and ZFHX3 (rs2106261) by real-time PCR.

**Results:**

The frequency of G/C in CDKN2B-AS1 (rs1333049) was higher in severe and moderate COVID-19 patients than in controls (71.1% and 53.3% vs. 37.8%). The frequency of the C/C of CDKN2B-AS1 (rs1333049) was higher in moderate COVID-19 patients than in controls (26.7% vs. 13.3%). There were no significant differences regarding genotype frequency and allelic distribution of ZFHX3 (rs2106261) between COVID-19 patients and healthy controls.

**Conclusion:**

CDKN2B-AS1 (rs1333049) gene polymorphism may play a role in determining the degree of COVID-19 severity. Further studies on its effect on cyclins and cyclin-dependent kinases (CDKs) [not measured in our study] may shed light on new treatment options for COVID-19.

**Supplementary Information:**

The online version contains supplementary material available at 10.1186/s12879-023-08564-7.

## Background

Coronavirus (CoV), which was well described in 1960, is a family of positive-strand enveloped RNA viruses that infect vertebrates [1].

The three known CoVs that cause severe diseases – severe acute respiratory syndrome-related coronavirus-1 (SARS-CoV-1) (the cause of SARS), Middle East respiratory syndrome coronavirus (MERS-CoV) and severe acute respiratory syndrome-related coronavirus-2 (SARS-CoV-2) – all come from bats [2].

SARS-CoV-2 is the causative organism of COVID-19 [3].

Researchers suggest that there is an intermediary, an animal that is infected by bats and carries the virus into humans. It is believed to be civet cats that are sold in live-animal markets in China in (special administrative regions) SARS [4].

Once the genetic material of SARS-CoV-2 enters the cell through receptor binding and then membrane fusion and penetration into the nucleus for replication, the virus controls the kinase family of enzymes and acts as switches that turn proteins on or off through the process of phosphorylation. The result is that the molecular machinery of the host is omitted to make new viral particles [1].

There are variations in the degree of severity of COVID-19, ranging from no symptoms and mild symptoms to intermediate care unit (IMCU) admission to intensive care unit (ICU) admission and mechanical ventilation [5].

SARS-CoV-2 patients with cardiovascular diseases, cerebrovascular diseases, chronic obstructive pulmonary diseases (COPDs), diabetes mellitus (DM), cancers, liver diseases, kidney diseases or obesity are at risk of a severe outcome compared with patients without such conditions [6], highlighting primarily the significant role of cardiovascular diseases as a risk factor for the development of severe or fatal acute COVID-19 [7, 8].

However, severe disease has been determined in patients who do not have the previously mentioned risk factors. Thus, we would like to review whether SNPs in definite genes could possibly have an impact on the degree of disease severity [9].

Several genetic studies have demonstrated an association between MI and genetic variants in a region on chromosome 9p21 [10] and in a region on chromosome 16q22 and AF and CI [11].

Recent genome-wide association studies (GWASs) on coronary artery disease (CAD) and MI implicated chromosome 9 on p21.3 locus in increasing susceptibility to MI [12].

Cyclin-dependent kinase inhibitor 2B antisense RNA 1 (CDKN2B-AS1) spans approximately 126.3 kb, overlaps with cyclin-dependent kinase inhibitor 2B (CDKN2B) (p15) at the 5´ end, comprises 20 exons that are prone to alternative splicing and is reported to be linked to CAD risk, hypertension (HTN) and stroke [13].

It was hypothesized that CDKN2B-AS1, coding for a long noncoding antisense RNA, was upregulated in atherosclerosis and can modulate atherosclerotic plaque progression. This occurs by downregulation of CDKN2B via RNA‒DNA triplex with the CDKN2B promoter to transfer CDKN2B-AS1-recruited EZH2 (for methylation of H3K27me3) to the promoter of the CDKN2B gene so that CTCF can bind to the promoter of the CDKN2B gene; thus, CDKN2B-AS1, by inhibiting macrophage reverse cholesterol transport, can promote the formation of atherosclerotic plaques [14].

CDKN2B encodes a cyclin-dependent kinase inhibitor that forms a complex with CDK 4/6 (necessary for progression of cell cycle) preventing activation by D-type cyclins or by CDK activating kinase (CAK) [15].

Because carriers of the 9p21 risk allele are known to have pathologically reduced expression of CDK inhibitors, treatment of primary smooth muscle cells (SMCs) with a specific inhibitor of CDK 4/6 prevented the upregulation of calcification markers [16].

Cyclins and CDKs are the main regulators of the progression of the cell cycle. Many viruses, including CoV, facilitate viral replication by regulating cell cycle progression through cyclin-CDK complexes [17].

Critically ill COVID-19 status is characterized by infiltration of the lungs with macrophages and neutrophils that cause diffuse lung alveolar damage, the histological equivalent to acute respiratory distress syndrome (ARDS). Neutrophils develop so-called neutrophil extracellular traps (NETs). However, ineffective clearance and dysregulation of NETosis result in pathological effects such as thrombo-inflammation. Inhibition of CDK4/6 blocks NET formation in a dose-responsive manner [18]. Selective inhibition of NETosis is a particularly attractive treatment because CDK6 inhibitors can prevent the cytokine storm and thus later ICU admission [18].

This is augmented by **Grinshpun et al.**.’s report that CDK4/6 inhibitor therapy of a breast cancer patient halted COVID-19 progression. On withdrawal of the drug, the full classic spectrum of illness appeared, including desaturation of oxygen necessitating ICU admission and mechanical ventilation [19].

In our study, we assessed the association of CDKN2B-AS1 (rs1333049) located on chromosome 9p21 and ZFHX3 (rs2106261) located on chromosome 16q22 SNPs with COVID-19 severity.

## Subjects and methods

### Subjects

This case‒controlled study was carried out in Medical Biochemistry & Molecular Biology and Anesthesiology, Intensive Care & Pain Management Departments, Faculty of Medicine, Menoufia University, and all patients were recruited from the ICU and IMCU, Menofia University Hospital. Ninety patients with severe COVID-19, 90 patients with moderate COVID-19 and 180 age- and gender-matched healthy controls were enrolled in this study from January 2022 to June 2022. Informed consent was obtained from all study participants and/or their legal guardians. The study design was approved by the appropriate ethics review board, followed the tenets of the Declaration of Helsinki and was approved by the ethics committee of the Faculty of Medicine, Menofia University (IRB number: 1/2022 BIO 40).

The inclusion criteria for patients were as follows: (1) Age ranging between 18 and 90 years old. (2) Patients with a history of HTN, DM, cardiovascular diseases, old stroke and COPD. (3) Diagnosis of COVID-19 was based on signs and symptoms and was confirmed by real-time PCR assay [20]. (4) Severe and moderate COVID-19 patients were recruited from the ICU and IMCU according to ABCD scoring for COVID-19 severity assessment [21].

The exclusion criteria for patients were as follows: (1) Patients with a current inflammatory or autoimmune disease. (2) Patients with recent surgery, trauma or heart attack. (3) Patients with a personal history of cancer. (4) Patients with a personal history of liver or kidney diseases. (5) Pregnant patients. (6) Previous severe or moderate SARS-CoV-2 infection.

### Methods

#### Sample collection

Nine milliliters (ml) of venous blood was withdrawn by venipuncture under complete aseptic conditions and was processed as follows: 2 ml was transferred into EDTA tubes for complete blood count (CBC), 2 ml was transferred into a blue-top tube containing 3.2% buffered sodium citrate for international normalized ratio (INR) and prothrombin concentration percentage (PC%) analysis, and 3 ml was transferred into a plain tube, left to clot for 15 min, and centrifuged for 10 min at 4000 rpm. The obtained serum was stored at -80 °C until other laboratory investigations were performed. The remaining 2 ml of blood was placed into EDTA-containing tubes for DNA extraction and genotyping of CDKN2B-AS1 (rs1333049) and ZFHX3 (rs2106261) SNPs by using the TaqMan allelic discrimination assay technique.

### Genotyping of CDKN2B-AS1 (rs1333049) and ZFHX3 (rs2106261) SNPs

#### 1-DNA extraction

DNA was extracted from whole blood by a GeneJET Genomic DNA Purification Kit (Thermo Scientific, Lithuania. cat# K0721) following the manufacturer’s protocol. DNA concentration, quality and purity were assessed using a Nanophotometer N-60 (Implen, Germany).

#### 2-Real time PCR

CDKN2B-AS1 (rs1333049) and ZFHX3 (rs2106261) gene polymorphisms were genotyped using an allelic discrimination assay by real-time PCR using a TaqMan probe (Applied Biosystems, Foster City, USA). Master Mix II (2x), primers and probes were supplied by Thermo Fisher Scientific. The probe sequence was labelled with [VIC/FAM] fluorescent dyes. The sequences of specific primers were as follows: **CDKN2B-AS1 (rs1333049)** [10]: forward primer: 5´CATACTAACCATATGATCAACAGTT 3´ and reverse primer: 5´AAAAGCAGCCACTCGCAGAGGTAAG 3´. **ZFHX3 (rs2106261)** [22]: forward primer: 5´ CGCGCCGAGGCCAACCATCCATTAAAATATCCAA 3´ and reverse primer 5´ ATGACGTGGCAGACTCAACCATCCATTAAAATATCCAAG 3´. Then, 12.5 µl of master mix was added to 1.25 µl of primer/probe mix and 6.25 µl of DNase-free water. Five microliters of genomic DNA extract for every sample and 5 µl of DNase-free water for the negative control reaction were applied. The cycling conditions were adjusted as follows: initial denaturation was performed at 95 °C for 10 min, followed by 50 cycles of denaturation at 95 °C for 15 s and 60 °C for 1 min for annealing and extension. Analysis of data was completed by a real-time PCR Instrument, Applied Biosystems®7500, software version 2.0.1.

### Statistical analysis

Analysis was achieved using version 20.0 of the IBM SPSS software package (Armonk, NY: IBM Corp). Qualitative data were described using numbers and percentages. We used the Kolmogorov‒Smirnov test to verify the normality of the distribution. Quantitative data were described using range (minimum and maximum), mean and standard deviation or median and interquartile range (IQR). We used the chi-square test for categorical variables to compare different groups and Fisher’s exact or Monte Carlo correction for chi-square when more than 20% of the cells had an expected count less than 5. We used Student’s t test for normally distributed quantitative variables to compare the two studied groups. We used the F test (ANOVA) for normally distributed quantitative variables to compare more than two groups and the post hoc test (Tukey) for pairwise comparisons. We used the Mann‒Whitney test for abnormally distributed quantitative variables to compare the two studied groups. We used the Kruskal‒Wallis test for abnormally distributed quantitative variables to compare more than two groups, and the post hoc test (Dunn’s test for multiple comparisons) was used for pairwise comparisons between each pair of groups. The population of the studied sample was explored to find its equilibrium with the Hardy-Weinberg equation. A P value of ≤ 0.05 was regarded as statistically significant.

## Results

### Demographic data of COVID-19 patients and healthy controls

There was a significant difference between severe and moderate COVID-19 cases regarding age (P1 < 0.001), but there was a nonsignificant difference between severe COVID-19 cases and healthy controls (P2 = 0.056) or between moderate COVID-19 cases and healthy controls (P3 = 0.115). There was a nonsignificant difference between COVID-19 patients and healthy controls according to sex (P = 0.108), and control individuals were chosen as age- and gender-matched. For severe and moderate COVID-19 patients, the mean age of severe COVID-19 patients was 65.46 ± 7.01 and that of moderate COVID-19 patients was 58.18 ± 17.36. The male gender in severe COVID-19 cases was 37.8% versus female gender 62.2%, and the male gender in moderate COVID-19 cases was 53.3% versus female gender 46.7%. The male gender in control individuals was 44.4% versus female gender 55.6% with a mean age of 61.56 ± 13.02. There was a significant difference between COVID-19 patients and healthy controls according to vaccination, with predominance in the control group (Table 1).

### Laboratory data of COVID-19 patients and healthy controls

There was a significant increase in WBC counts in severe and moderate COVID-19 patients compared with controls (P2 < 0.001, P3 < 0.001). There was a significant decrease in platelet count in severe and moderate COVID-19 patients compared with controls and in severe COVID-19 patients compared to moderate COVID-19 patients (P1 = 0.03, P2 < 0.001, P3 < 0.001). There was a significant decrease in HB in severe and moderate COVID-19 patients compared with controls and in severe COVID-19 patients compared to moderate COVID-19 patients (P < 0.001 each).

Regarding liver function tests, there was a significant increase in SGOT in severe and moderate COVID-19 patients compared with controls (P2 < 0.001, P3 < 0.001), while there was a significant increase in SGPT in moderate COVID-19 patients compared to severe COVID-19 patients (P1 = 0.011).

Regarding kidney function tests, there was a significant increase in BUN in severe and moderate COVID-19 patients compared with controls (P2 < 0.001, P3 < 0.001) and in severe COVID-19 patients compared to moderate COVID-19 patients (P1 = 0.019). There was a significant increase in creatinine in severe COVID-19 patients compared with moderate COVID-19 patients (P1 < 0.001) and controls (P2 < 0.001). There was a significant decrease in Na + levels in moderate COVID-19 patients compared with severe COVID-19 patients (P1 < 0.001) and a significant increase in K + levels in severe COVID-19 patients compared to controls (P2 < 0.001).

Regarding the lipid profile, there was a significant increase in TGs (P2 < 0.001, P3 = 0.001) and a significant decrease in total cholesterol, HDL and LDL (P2 < 0.001, P3 < 0.001 each) in severe and moderate COVID-19 patients compared with controls.

There was a significant decrease in PC% in severe and moderate COVID-19 patients compared with controls and in severe COVID-19 patients compared to moderate COVID-19 patients (P < 0.001) and a significant increase in INR in severe and moderate COVID-19 patients compared with controls (P2 < 0.001, P3 < 0.001).

Finally, there was a significant increase in CRP, ferritin and D-dimer in severe and moderate COVID-19 patients compared with controls (P2 < 0.001, P3 < 0.001) and a significant increase in CRP (P1 = 0.044) and ferritin (P1 = 0.03) in severe COVID-19 patients compared to moderate COVID-19 patients (Table 1).

**Table 1:** Demographic and laboratory data of COVID-19 patients and healthy controls

	Severe COVID-19 cases (n = 90)	Moderate COVID-19 cases (n = 90)	Healthy controls (n = 180)	Test of sig.	P
**Gender**					
Male	34 (37.8%)	48 (53.3%)	80 (44.4%)	χ^2^ = 4.444	0.108
Female	56 (62.2%)	42 (46.7%)	100 (55.6%)		
**Age (years)**					
Mean ± SD.	65.46 ± 7.01	58.18 ± 17.36	61.56 ± 13.02	F = 14.319^*^	< 0.001^*^
**Sig.bet.Grps**	P1 < 0.001^*^,P2 = 0.056,P3 = 0.115				
**Vaccine**					
Yes	4	2	104	χ^2^=125.830	< 0.001^*^
No	86	88	76		
**WBCs** 4.00–11/(mcL)					
Median (IQR)	10.0 (7.50–13.20)	10.90 (7.50–14.50)	8.55 (6.90–9.30)	H = 40.927^*^	< 0.001^*^
**Sig.bet.Grps**	P1 = 0.685,P2 < 0.001^*^,P3 < 0.001^*^				
**Platelet** 150–400/(mcL)					
Median (IQR)	205.0 (152.0–249.0)	231.0 (168.0–311.0)	313.50 (278.0–364.0)	H = 90.737^*^	< 0.001^*^
**Sig.bet.Grps**	P1 = 0.030^*^,P2 < 0.001^*^,P3 < 0.001^*^				
**HB (g/dl)** Male: 14–18 g/dL Female: 12–16 g/dL					
Mean ± SD.	10.64 ± 1.87	11.56 ± 2.13	12.89 ± 1.42	F = 53.898^*^	< 0.001^*^
**Sig.bet.Grps**	P1 < 0.001^*^,P2 < 0.001^*^,P3 < 0.001^*^				
**SGOT (U/L)** 8–45 (U/L)					
Median (IQR)	37.0 (25.0–63.0)	38.0 (29.0–65.0)	24.0 (19.0–31.0)	H = 72.280^*^	< 0.001^*^
**Sig.bet.Grps**	P1 = 0.682,P2 < 0.001^*^,P3 < 0.001^*^				
**SGPT (U/L)** 7–56 (U/L)					
Median (IQR)	27.0 (18.0–38.0)	33.0 (21.0–51.0)	31.0 (23.0–24.0)	H = 6.769^*^	0.034^*^
	P1 = 0.011^*^,P2 = 0.057,P3 = 0.301				
**BUN (mg/dL)** 6–24 mg/dL					
Median (IQR)	45.0 (22.0–73.0)	27.0 (18.0–41.0)	20.0 (17.0–22.0)	H = 91.340^*^	< 0.001^*^
**Sig.bet.Grps**	P1 = 0.0190^*^,P2 < 0.001^*^,P3 < 0.001^*^				
**Creatinine (mg/dL)** Male: 0.6-1.2 mg/dL Female: 0.5 to 1.1 mg/dL					
Median (IQR)	1.30 (0.85–1.94)	0.90 (0.70–1.50)	1.00 (0.90–1.10)	H = 22.511^*^	< 0.001^*^
**Sig.bet.Grps**	P1 < 0.001^*^,P2 < 0.001^*^,P3 = 0.808				
**Na+ (mEq/L)** 135–145 (mEq/L)					
Mean ± SD.	140.19 ± 5.45	138.09 ± 5.75	139.27 ± 2.80	F = 5.100^*^	< 0.001^*^
**Sig.bet.Grps**	P1 < 0.001^*^,P2 = 0.242, P3 = 0.242				
** K+ (mEq/L)** 3.5–5.3 (mEq/L)					
Mean ± SD.	4.45 ± 0.70	4.23 ± 0.71	4.14 ± 0.53	F = 7.298^*^	< 0.001^*^
**Sig.bet.Grps**	P1 = 0.070,P2 < 0.001^*^,P3 = 0.518				
**TG (mg/dL)** Male: 40–160 mg/dL Female: 35–135 mg/dL					
Median (IQR)	130.0 (109.0–138.0)	124.0 (104.0–162.0)	108.0 (102.50–123.0)	H = 41.027^*^	< 0.001^*^
**Sig.bet.Grps**	P1 = 0.269,P2 < 0.001^*^,P3 = 0.001^*^				
**Cholesterol (mg/dL)** 125–200 mg/dL					
Median (IQR)	117.0 (94.0–134.0)	110.0 (98.0–133.0)	158.50 (149.0–172.0)	H = 182.552^*^	< 0.001^*^
**Sig.bet.Grps**	P1 = 0.436, P2 < 0.001^*^,P3 < 0.001^*^				
**HDL (mg/dL)** Male: 35–55 mg/dL Female: 45–65 mg/dL					
Median (IQR)	35.0 (30.0–42.50)	33.50 (28.0–42.0)	49.15 (45.80–55.90)	H = 145.707^*^	< 0.001^*^
**Sig.bet.Grps**	P1 = 0.981, P2 < 0.001^*^, P3 < 0.001^*^				
**LDL (mg/dL)** < 130 mg/dL					
Median (IQR)	48.0 (38.46–68.40)	50.40 (37.76–63.0)	88.0 (77.78–94.60)	H = 161.798^*^	< 0.001^*^
**Sig.bet.Grps**	P1 = 0.551, P2 < 0.001^*^, P3 < 0.001^*^				
**PC%**					
Mean ± SD.	73.29 ± 17.8946	78.78 ± 16.8114	96.51 ± 2.8254	F = 126.690^*^	< 0.001^*^
**Sig.bet.Grps**	P1 < 0.001^*^,P2 < 0.001^*^,P3 < 0.001^*^				
**INR** 0.9–1.1					
Median (IQR)	1.20 (1.10–1.40)	1.16 (1.05–1.30)	1.03 (1.0–1.06)	H = 125.254^*^	< 0.001^*^
**Sig.bet.Grps**	P1 = 0.130, P2 < 0.001^*^, P3 < 0.001^*^				
**CRP (mg/L)** < 3 mg/L					
Median (IQR)	60.0 (24.0–96.0)	48.0 (17.0–94.0)	4.50 (2.0–7.0)	H = 194.747^*^	< 0.001^*^
**Sig.bet.Grps**	P1 = 0.044^*^,P2 < 0.001^*^,P3 < 0.001^*^				
**Ferritin (µg/L)** Male: 24–336 (µg/L) Female: 11–307 (µg/L)					
Median (IQR)	445.0 (294.0–700.0)	420.0 (214.0–657.0)	93.0 (88.0–100.0)	H = 214.915^*^	< 0.001^*^
**Sig.bet.Grps**	P1 = 0.030^*^,P2 < 0.001^*^,P3 < 0.001^*^				
D- **dimer (µg/mL)** 0-0.50 (µg/mL)					
Median (IQR)	0.94 (0.80–1.54)	0.81 (0.6–2.20)	0.20 (0.15–0.23)	H = 243.755^*^	< 0.001^*^
**Sig.bet.Grps**	P1 = 0.077,P2 < 0.001^*^,P3 < 0.001^*^				

IQR: **Interquartile range** SD: **Standard deviation**

χ^2^: **Chi square test**

F: F for **one-way ANOVA test**, pairwise comparison bet. each 2 groups was performed using **a post hoc test (Tukey)**

H: H for **Kruskal–Wallis test**, Pairwise comparison bet. each 2-group analysis was performed using **a post hoc test (Dunn’s test for multiple comparisons)**

*: Statistically significant at p ≤ 0.05

P1: p value for comparing severe and moderate COVID-19 cases

P2: p value for comparing severe COVID-19 cases and healthy controls

P3: p value for comparing moderate COVID-19 cases and healthy controls

**WBCs**: White blood cells **HB**: Hemoglobin

**SGOT**: Serum glutamic-oxaloacetic transaminase **SGPT**: Serum glutamic-pyruvic transaminase

**BUN**: Blood urea nitrogen **TG**: Triglyceride

**HDL**: High-density lipoprotein **LDL**: Low-density lipoprotein

**PC%**: prothrombin concentration percentage **INR**: International normalized ratio

**CRP**: c-reactive protein

### CDKN2B-AS1 (rs1333049) and ZFHX3 (rs2106261) gene polymorphisms of COVID-19 patients and healthy controls

There was a significant difference regarding the genotype frequency and allelic distribution of CDKN2B-AS1 (rs1333049) between COVID-19 patients and healthy controls (P < 0.001). The frequency of the GC genotype was significantly higher in severe COVID-19 cases (71.1%) and moderate COVID-19 cases (53.3%) than in healthy controls (37.8%), and the frequency of the CC genotype was significantly higher in moderate COVID-19 cases (26.7%) than in healthy controls (13.3%). There was no significant difference regarding genotype frequency (MCp = 0.253) and allelic distribution (P = 0.319) of ZFHX3 (rs2106261) between COVID-19 patients and healthy controls (Table 2).

**Table 2:** CDKN2B-AS1 (rs1333049) and ZFHX3 (rs2106261) gene polymorphisms of COVID-19 patients and healthy controls

	Severe COVID-19 cases (n = 90)		Moderate COVID-19 cases (n = 90)		Healthy controls (n = 180)		χ^2^	p
	No.	%	No.	%	No.	%		
**CDKN2B-AS1 (rs1333049)**								
G/G	12	13.3	18	20.0	88	48.9	49.106^*^	< 0.001^*^
G/C	64	71.1	48	53.3	68	37.8		
C/C	14	15.6	24	26.7	24	13.3		
^**HW**^ **χ2 (p)**	**16.098(< 0.001** ^*^ **)**		**0.459(0.498)**		**3.286(0.070)**			
**Allele**								
G	88	48.9	84	46.7	244	67.78	29.696^*^	< 0.001^*^
C	92	51.1	96	53.3	116	32.22		
**ZFHX3 (rs2106261)**								
C/C	56	62.2	66	73.3	128	71.1	5.3884	MCp = 0.253
C/T	34	37.8	24	26.7	50	27.8		
T/T	0	0.0	0	0.0	2	1.1		
^**HW**^ **χ2 (p**_**0**_ **)**	**4.881(0.027** ^*^ **)**		**2.130(0.144)**		**1.436(0.231)**			
**Allele**								
C	146	81.1	156	86.7	306	85.0	2.2838	0.319
T	34	18.9	24	13.3	54	15.0		

χ^2^: **Chi square test** MC: **Monte Carlo**

p: p value for comparing COVID-19 patients and healthy controls

*: Statistically significant at p ≤ 0.05

HWχ2 (p0): Chi square for goodness of fit for Hardy-Weinberg equilibrium (If P < 0.05 - not consistent with HWE)

**Fig. 1:**
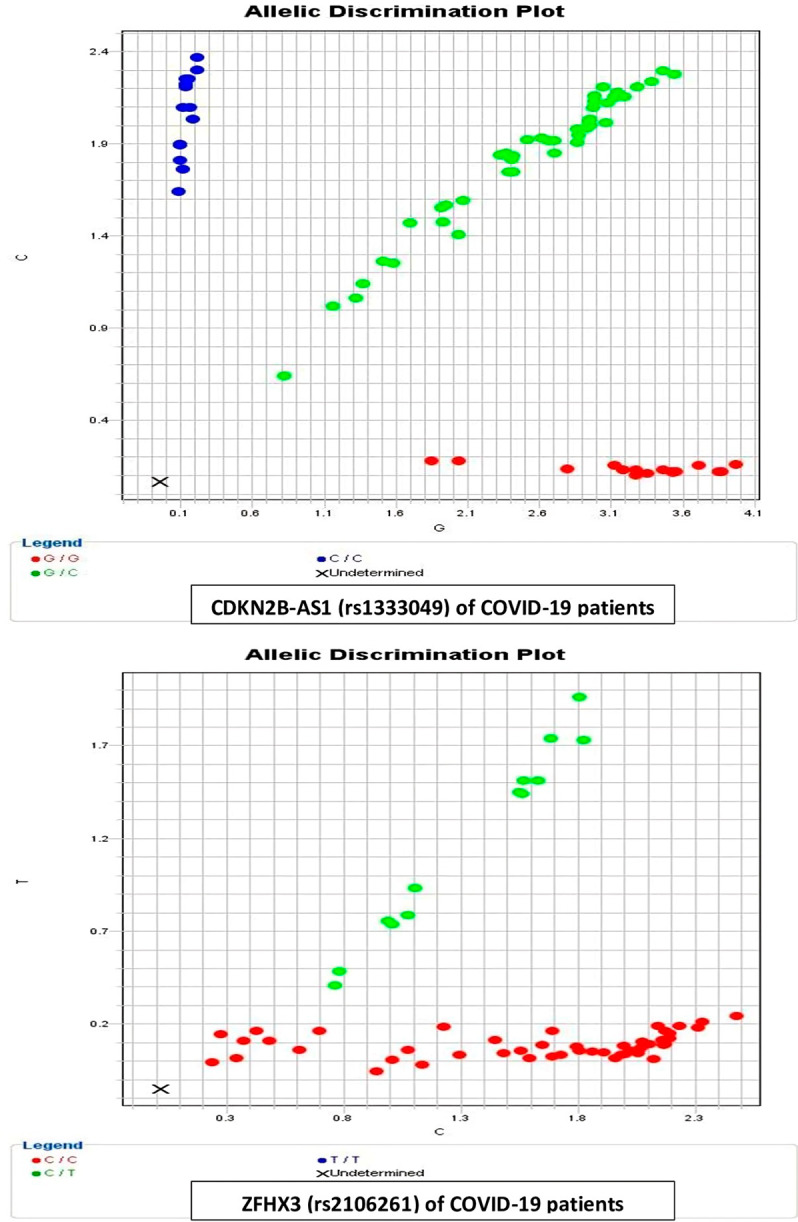
Allelic discrimination plot of CDKN2B-AS1 (rs1333049) and ZFHX3 (rs2106261) in COVID-19 patients

An allelic discrimination plot of CDKN2B-AS1 (rs1333049) and ZFHX3 (rs2106261) in COVID-19 patients is shown in Fig. 1.

### CDKN2B-AS1 (rs1333049) and ZFHX3 (rs2106261) gene polymorphisms of COVID-19 patients and healthy controls in different models

Regarding CDKN2B-AS1 (rs1333049), a significantly higher prevalence of the genotype (GC) and the variant allele (C) in severe COVID-19 patients and moderate COVID-19 patients compared to the control group was observed under the dominant, codominant−1 and overdominant modes of inheritance and in severe COVID-19 patients compared to moderate COVID-19 patients under the overdominant mode of inheritance. The significantly higher prevalence of the genotype (CC) in moderate COVID-19 patients compared to the control group was observed under the recessive and codominant−2 modes of inheritance and in severe COVID-19 patients under the codominant-2 mode of inheritance. Regarding ZFHX3 (rs2106261), there was a nonsignificant difference between the three studied groups under the dominant, recessive, codominant−1, codominant−2 and overdominant modes of inheritance (Table 3).

**Table 3:** CDKN2B-AS1 (rs1333049) and ZFHX3 (rs2106261) gene polymorphisms of COVID-19 patients and healthy controls in different models

	Ia vs. Ib®		Ia vs. II®		Ib vs. II®	
	p	OR(95% C.I)	p	OR(95% C.I)	p	OR(95% C.I)
**CDKN2B-AS1 (rs1333049)**						
GG® vs. GC + CC(Dominant)	0.233	1.625(0.732–3.608)	< 0.001^*^	6.217(3.167–12.204)	< 0.001^*^	3.826(2.113–6.926)
GG + GC® vs. CC (Recessive)	0.071	0.507(0.242–1.059)	0.621	1.197(0.586–2.445)	0.007^*^	2.364(1.253–4.459)
GG vs. GC ® (Codominant-1)	0.098	2.0(0.880–4.544)	< 0.001^*^	6.902(3.451–13.803)	< 0.001^*^	3.451(1.843–6.463)
GG vs. CC® (Codominant-2)	0.790	0.875(0.327– 2.341)	0.001*	4.278(1.751–10.453)	< 0.001^*^	4.889(2.287–10.451)
GC vs. GG + CC® (overdominant)	0.014^*^	0.464(0.251– 0.860)	< 0.001^*^	0.247(0.143–0.426)	0.02^*^	0.531(0.318–0.887)
**ZFHX3 (rs2106261)**						
CC® vs. CT + TT (Dominant)	0.112	1.670(0.887–3.142)	0.141	1.495(0.876–2.550)	0.702	0.895(0.507–1.579)
CC + CT ® vs. TT (Recessive)	1.000	–	0.549	0.395(0.019–8.304)	0.549	0.395(0.019–8.304)
CC® vs. CT (Codominant-1)	0.112	1.670(0.887–3.142)	0.108	1.554(0.908–2.659)	0.806	0.931(0.526–1.646)
CC® vs. TT (Codominant-2)	0.935	1.177(0.023–60.278)	0.613	0.455(0.022–9.629)	0.541	0.387(0.018–8.167)
CT vs. CC + TT ® (overdominant)	0.112	0.599(0.318–1.127)	0.095	0.634(0.370–1.083)	0.847	1.058(0.598–1.870)

OR: Odds ratio for CI: confidence interval ®: reference group

p: p value for Chi square test for comparing COVID-19 patients and healthy controls

*: Statistically significant at p ≤ 0.05

### Relation between CDKN2B-AS1 (rs1333049) gene polymorphism and laboratory data and prognosis in severe COVID-19 patients

Regarding the relation of CDKN2B-AS1 (rs1333049) genotype distribution in severe COVID-19 patients, there was a significant association of lower WBCs (P < 0.001), lower platelet count (P < 0.001), lower HDL (P = 0.027) and higher ferritin level (P < 0.001) in G/C and C/C genotypes compared to the G/G genotype. There was also a significant association of higher D-dimer (P = 0.016) in the G/C genotype. There was no significant association regarding other parameters (Table 4).

**Table 4:** Relation between CDKN2B-AS1 (rs1333049) gene polymorphism and laboratory data and prognosis in severe COVID-19 cases (n = 90)

	Reference range	CDKN2B-AS1 (rs1333049)			Test of sig.	p
		G/G (n = 12)	G/C (n = 64)	C/C (n = 14)		
**WBCs**	4.00–11/(mcL)					
Median		25.50	10.50	7.40	H = 15.890^*^	< 0.001^*^
**Platelet**	150–400/(mcL)					
Median		298.0	190.0	219.0	H = 21.249^*^	< 0.001^*^
**HB (g/dl)**	Male: 14–18 g/dL					
Mean ± SD.	Female: 12–16 g/dL	10.03 ± 1.33	10.77 ± 1.93	10.60 ± 2.22	F = 0.779	0.462
**SGOT (U/L)**	8–45 (U/L)					
Median		42.0	37.0	37.0	H = 0.811	0.667
**SGPT (U/L)**	7–56 (U/L)					
Median		27.50	26.50	17.0	H = 1.792	0.408
**BUN (mg/dL)**	6–24 mg/dL					
Median		42.0	47.50	49.0	H = 0.451	0.798
**Creatinine (mg/dL)**	Male: 0.6-1.2 mg/dL					
Median	Female: 0.5 to 1.1 mg/dL	1.35	1.28	1.60	H = 0.083	0.959
**Na** ^**+**^ **(mEq/L)**	135–145 (mEq/L)					
Mean ± SD.		141.50 ± 4.76	139.84 ± 5.79	140.63 ± 5.02	F = 0.516	0.599
** K** ^**+**^ **(mEq/L)**	3.5–5.3 (mEq/L)					
Mean ± SD.		4.47 ± 0.75	4.41 ± 0.72	4.61 ± 0.63	F = 0.495	0.611
**TG (mg/dL)**	Male: 40–160 mg/dL					
Median	Female: 35–135 mg/dL	144.50	130.0	114.0	H = 4.067	0.131
**Cholesterol (mg/dL)**	125–200 mg/dL					
Median		122.50	116.0	107.50	H = 0.325	0.850
**HDL (mg/dL)**	Male: 35–55 mg/dL					
Median	Female: 45–65 mg/dL	41.25	35.50	28.50	H = 7.224^*^	0.02^*^7
**LDL (mg/dL)**	< 130 mg/dL					
Median		48.50	46.30	48.92	H = 0.435	0.805
**PC%**						
Mean ± SD.		72.85 ± 19.02	73.71 ± 18.47	71.76 ± 15.15	F = 0.071	0.931
**INR**	0.9–1.1					
Median		1.25	1.18	1.20	H = 1.838	0.399
**CRP (mg/L)**	< 3 mg/L					
Median		48	54.5	78.0	H = 1.599	0.450
**Ferritin (µg/L)**	Male: 24–336 (µg/L)					
Median	Female: 11–307 (µg/L)	225.0	538.50	380.0	H = 17.033^*^	< 0.001^*^
**D-dimer (µg/mL)**	0-0.50 (µg/mL)					
Median		0.99	1.04	0.70	H = 8.32^*^2	0.0^*^16
**Prognosis**						
Improved		2 (16.7%)	22 (34.4%)	4 (28.6%)	χ^2^=1.529	0.466
Death		10 (83.3%)	42 (65.6%)	10 (71.4%)		

IQR: **Interquartile range** SD: **Standard deviation**

F: F **for one-way ANOVA test** H: H for **Kruskal–Wallis test**

χ2: **Chi square test**

p: p value for comparison between the studied categories

*: Statistically significant at p ≤ 0.05

**WBCs**: White blood cells **HB**: Hemoglobin

**SGOT**: Serum glutamic-oxaloacetic transaminase **SGPT**: Serum glutamic-pyruvic transaminase

**BUN**: Blood urea nitrogen **TG**: Triglyceride

**HDL**: High-density lipoprotein **LDL**: Low-density lipoprotein

**PC%**: prothrombin concentration percentage **INR**: International normalized ratio

**CRP**: c-reactive protein

### Relation between CDKN2B-AS1 (rs1333049) gene polymorphism and laboratory data and prognosis in moderate COVID-19 patients

Regarding the relation of CDKN2B-AS1 (rs1333049) genotype distribution in moderate COVID-19 patients, there was a significant association of lower platelet count (P < 0.001), lower Hb (P = 0.012), higher CRP (P = 0.048) and higher ferritin (P = 0.019) in G/C and C/C genotypes compared to the G/G genotype. There was a significant association of lower PC% (P < 0.001) and prolonged INR (P < 0.001) in the G/C genotype, while there was no significant association regarding other parameters (Table 5).

**Table 5:** Relation between CDKN2B-AS1 (rs1333049) gene polymorphism and laboratory data and prognosis in moderate COVID-19 cases (n = 90)

	Reference range	CDKN2B-AS1 (rs1333049)			Test of sig.	p
		G/G (n = 18)	G/C (n = 48)	C/C (n = 24)		
**WBCs**	4.00–11/(mcL)					
Median		8.20	11.0	11.20	H = 2.058	0.357
**Platelet**	150–400/(mcL)					
Median		318.0	215.50	207.0	H = 16.271^*^	< 0.001^*^
**HB (g/dl)**	Male: 14–18 g/dL					
Mean ± SD.	Female: 12–16 g/dL	12.14 ± 2.24	11.88 ± 2.17	10.48 ± 1.61	F = 4.64^*^1	0.012^*^
**SGOT (U/L)**	8–45 (U/L)					
Median		29.0	38.0	45.0	H = 4.254	0.119
**SGPT (U/L)**	7–56 (U/L)					
Median		30.0	34.0	29.50	H = 0.983	0.612
**BUN (mg/dL)**	6–24 mg/dL					
Median		26.0	32.50	22.0	H = 1.238	0.539
**Creatinine (mg/dL)**	Male: 0.6-1.2 mg/dL					
Median	Female: 0.5 to 1.1 mg/dL	0.88	1.05	0.75	H = 5.451	0.066
**Na** ^**+**^ **(mEq/L)**	135–145 (mEq/L)					
Mean ± SD.		139.22 ± 5.37	138.29 ± 5.96	136.83 ± 5.62	F = 0.950	0.391
** K** ^**+**^ **(mEq/L)**	3.5–5.3 (mEq/L					
Mean ± SD.		4.44 ± 0.68	4.14 ± 0.78	4.26 ± 0.54	F = 1.214	0.302
**TG (mg/dL)**	Male: 40–160 mg/dL					
Median	Female: 35–135 mg/dL	108.0	117.0	150.0	H = 4.143	0.126
**Cholesterol (mg/dL)**	125–200 mg/dL					
Median		111.0	102.50	114.50	H = 1.248	0.536
**HDL (mg/dL)**	Male: 35–55 mg/dL					
Median	Female: 45–65 mg/dL	33.50	34.0	34.0	H = 1.012	0.603
**LDL (mg/dL)**	< 130 mg/dL					
Median		54.60	48.10	52.50	H = 2.403	0.301
**PC%**						
Mean ± SD.		79.03 ± 15.05	74.11 ± 17.80	87.92 ± 12.10	F = 6.007	< 0.001^*^
**INR**	0.9–1.1					
Median		1.16	1.20	1.05	H = 14.456^*^	< 0.001^*^
**CRP (mg/L)**	< 3 mg/L					
Median		17.0	55.0	24.0	H = 6.043^*^	0.048^*^
**Ferritin (µg/L)**	Male: 24–336 (µg/L)					
Median	Female: 11–307 (µg/L)	250.0	523.0	305.0	H = 7.892*	0.019^*^
**D-dimer (µg/mL)**	0-0.50 (µg/mL)					
Median		0.63	0.80	0.90	H = 1.1.474	0.479
**Prognosis**						
Improved		12 (66.7%)	26 (54.2%)	16 (66.7%)	χ^2^=1.458	0.482
Death		6 (33.3%)	22 (45.8%)	8 (33.3%)		

IQR: **Interquartile range** SD: **Standard deviation**

F: F **for one-way ANOVA test** H: H for **Kruskal–Wallis test**

χ2: **Chi square test**

p: p value for comparison between the studied categories

*: Statistically significant at p ≤ 0.05

**WBCs**: White blood cells **HB**: Hemoglobin

**SGOT**: Serum glutamic-oxaloacetic transaminase **SGPT**: Serum glutamic-pyruvic transaminase

**BUN**: Blood urea nitrogen **TG**: Triglyceride

**HDL**: High-density lipoprotein **LDL**: Low-density lipoprotein

**PC%**: prothrombin concentration percentage **INR**: International normalized ratio

**CRP**: c-reactive protein

### Relation between ZFHX3 (rs2106261) gene polymorphism and laboratory data and prognosis in severe COVID-19 patients

There was a significant association of higher creatinine (P = 0.024), lower LDL (P = 0.015) and a higher incidence of death (P = 0.032) in the C/T genotype compared to the C/C genotype, while there was no significant association regarding other parameters (Table 6).

**Table 6:** Relation between ZFHX3 (rs2106261) gene polymorphism and laboratory data and prognosis in severe COVID-19 cases (n = 45)

	Reference range	ZFHX3 (rs2106261)		Test of sig.	p
		C/C (n = 56)	C/T (n = 34)		
**WBCs**	4.00–11/(mcL)				
Median		11.0	8.70	U = 788.0	0.172
**Platelet**	150–400/(mcL)				
Median		198.0	209.0	U = 936.0	0.894
**HB (g/dl)**	Male: 14–18 g/dL				
Mean ± SD.	Female: 12–16 g/dL	10.79 ± 1.89	10.41 ± 1.85	t = 0.940	0.350
**SGOT (U/L)**	8–45 (U/L)				
Median		36.50	50.0	U = 824.0	0.286
**SGPT (U/L)**	7–56 (U/L)				
Median		27.50	19.0	U = 776.0	0.143
**BUN (mg/dL)**	6–24 mg/dL				
Median		38.0	49.0	U = 820.0	0.272
**Creatinine (mg/dL)**	Male: 0.6-1.2 mg/dL				
Median	Female: 0.5 to 1.1 mg/dL	1.04	1.50	U = 682.0^*^	0.024^*^
**Na** ^**+**^ **(mEq/L)**	135–145 (mEq/L)				
Mean ± SD.		139.98 ± 3.82	140.53 ± 7.45	t = 0.463	0.645
** K** ^**+**^ **(mEq/L)**	3.5–5.3 (mEq/L				
Mean ± SD.		4.38 ± 0.69	4.57 ± 0.72	t = 1.295	0.199
**TG (mg/dL)**	Male: 40–160 mg/dL				
Median	Female: 35–135 mg/dL	130.0	130.0	U = 932.0	0.868
**Cholesterol (mg/dL)**	125–200 mg/dL				
Median		126.0	107.50	U = 724.0	0.0577
**HDL (mg/dL)**	Male: 35–55 mg/dL				
Median	Female: 45–65 mg/dL	35.0	35.0	U = 902.0	0.677
**LDL (mg/dL)**	< 130 mg/dL				
Median		53.95	40.60	U = 660.0^*^	0.015^*^
**PC%**					
Mean ± SD.		73.24 ± 19.25	73.38 ± 15.67	t = 0.037	0.970
**INR**	0.9–1.1				
Median		1.15	1.20	U = 900.0	0.664
**CRP (mg/L)**	< 3 mg/L				
Median		54.50	78.0	U = 856.0	0.423
**Ferritin (µg/L)**	Male: 24–336 (µg/L)				
Median	Female: 11–307 (µg/L)	447.50	440.0	U = 880.0	0.549
**D-dimer (µg/mL)**	0-0.50 (µg/mL)				
Median		1.04	0.90	U = 952.0	1.000
**Prognosis**					
Improved		22 (39.3%)	6 (17.6%)	χ^2^=4.622^*^	0.032^*^
Death		34 (60.7%)	28 (82.4%)		

SD: **Standard deviation** χ2: **Chi square test**

t: **Student’s t test** U: **Mann–Whitney test**

p: p value for comparison between the studied categories

*: Statistically significant at p ≤ 0.05

**WBCs**: White blood cells **HB**: Hemoglobin

**SGOT**: Serum glutamic-oxaloacetic transaminase **SGPT**: Serum glutamic-pyruvic transaminase

**BUN**: Blood urea nitrogen **TG**: Triglyceride

**HDL**: High-density lipoprotein **LDL**: Low-density lipoprotein

**PC%**: prothrombin concentration percentage **INR**: International normalized ratio

**CRP**: c-reactive protein

## Discussion

As the COVID-19 pandemic creates a worldwide crisis of overwhelming morbidity and mortality [23], it is imperative and urgent to provide insights into the association between host genetic factors and clinical outcomes.

Our study revealed that the frequency of the G/C genotype regarding CDKN2B-AS1 (rs1333049) was significantly higher in severe and moderate COVID-19 patients than in the control group, and the frequency of the C/C genotype was significantly higher in moderate COVID-19 patients than in the control group. This may explain the ability of coronaviruses to replicate efficiently and cause severe COVID-19 in patients with this SNP.

These results are in accordance with the significant role of comorbidities, primarily cardiovascular disease, as a risk factor for the development of severe or fatal acute COVID-19 [7, 8]. These results are in accordance with **Yahia et al.** [10], who demonstrated that the frequency of the G/C and C/C genotypes of CDKN2B-AS1 (rs1333049) was higher in acute MI patients than in controls (54% vs. 36% and 36% vs. 18%, respectively, P < 0.001), while controls showed an increased frequency of the G/G genotype (46% vs. 10%, P < 0.001). Additionally, **Kaur et al.** [13] stated that the G/C genotype was highly prevalent among patients with acute MI compared to controls (62.8% vs. 34.0%, P = 0.002), while the G/G genotype had a higher frequency in controls than in cases (16.8% vs. 4.0%).

In our study, there was a significant increase in WBC counts in severe and moderate COVID-19 patients compared with controls. According to **Palladino** [24], leukocytes and neutrophils were significantly higher in severe COVID-19 patients than in nonsevere patients. Furthermore, along with the progression of COVID-19, both leukocytes and neutrophils increased in severe groups [25]. According to **Zhu et al.** [26], patients with higher WBC counts were at a high risk of death.

We found that there was a significant decrease in platelet count in severe COVID-19 patients compared with moderate COVID-19 patients and controls. **Lippi et al.** [27] correlated that a decrease in platelet count was associated with more severe COVID-19 and higher mortality. In fact, most thrombocytopenic patients have altered coagulation parameters with high concentrations of D-dimer, which confirms the theory of triggering intravascular coagulation [28].

Our study revealed that there was a significant decrease in HB in severe COVID-19 patients compared with moderate COVID-19 patients and controls. Accordingly, **Wang et al.** [29] demonstrated a reduction in haemoglobin concentration in 19% of hospital-admitted patients, **while Huang et al.** [30] demonstrated reduced haemoglobin concentrations in 38% of hospitalized patients.

Regarding liver function tests, we found that there was a significant increase in SGOT in severe and moderate COVID-19 patients compared with controls without a significant increase in SGPT. The significant increase in SGOT without affecting SGPT can indicate cardiac complications. This is consistent with **Pazgan-Simon et al.** [31], who postulated that it seems that liver injury in COVID-19 patients with no underlying liver disease was not associated with a higher risk of mortality and that liver injury secondary to COVID-19 was mild.

Regarding kidney function tests, we found that there was a significant increase in BUN and creatinine levels in severe and moderate COVID-19 patients compared with controls and in BUN in severe COVID-19 patients compared to moderate COVID-19 patients. This matched with **Liu et al.** [32], who found that the prevalence of elevated BUN and serum creatinine was 6.29% and 5.22%, respectively, at admission, and elevated baseline levels of BUN and serum creatinine were associated with increased all-cause mortality risk. Additionally, we found an increase in K + levels in severe COVID-19 patients compared with controls. These results are in accordance with **Amin et al.** [33], who postulated that hyperkalaemic patients were more likely to be older and that hyperkalaemic patients were more likely to have a history of DM, MI and COPD than nonhyperkalaemic patients. Additionally, we found a decrease in Na + levels in moderate COVID-19 patients compared to severe COVID-19 patients. This matched with **Islam et al.** [34], who postulated that moderate COVID-19 patients had 2.15 (1.04–4.5) times higher odds of suffering from hyponatremia.

Regarding the lipid profile, we found that there was a significant increase in TGs and a significant decrease in total cholesterol, HDL and LDL in severe and moderate COVID-19 patients compared with controls. Additionally, there was a significant association of lower LDL in severe COVID-19 patients regarding the C/T genotype of ZFHX3 (rs210626) genotype distribution and lower HDL in severe COVID-19 patients regarding the G/C and C/C genotypes of CDKN2B-AS1 (rs1333049) genotype distribution. This is in accordance with a meta-analysis of nineteen studies and twenty-two studies that stated that decreased levels of total cholesterol, LDL and HDL were associated with severity and mortality in patients with COVID-19 [35, 36]. Serum triglyceride levels in patients with COVID-19 were variable, and elevated serum triglyceride levels were reported in mild or severe COVID-19 patients but not in patients with critical disease (septic shock and respiratory or multiple organ failure) [37].

Our study found that there was a significant decrease in PC% and a significant increase in INR in severe and moderate COVID-19 patients compared with controls and in PC% in severe COVID-19 patients compared to moderate COVID-19 patients. Additionally, there was a significant association between lower PC% and prolonged INR in the G/C genotype regarding the SNP rs1333049 genotype distribution of CDKN2B-AS1 in moderate COVID-19 patients. This is consistent with **Zinellu et al.** [38], who postulated that INR prolongation was significantly associated with COVID-19 severity and mortality.

Additionally, our study demonstrated that there was a significant increase in CRP, ferritin and D-dimer in severe and moderate COVID-19 patients compared with controls and in CRP and ferritin in severe COVID-19 patients compared to moderate COVID-19 patients. Additionally, there was a significant association of D-dimer in severe COVID-19 patients, higher CRP in moderate COVID-19 patients and higher ferritin levels in both groups when comparing G/C and C/C genotypes with the G/G genotype regarding the relation of CDKN2B-AS1 (rs1333049) genotype distribution. **Liu et al.** [39] postulated that higher levels of CRP, ferritin and D-dimer were associated with poor COVID-19 outcomes.

Surprisingly, there was a significant association of a higher incidence of death in the C/T genotype in severe COVID-19 patients regarding the ZFHX3 (rs2106261) genotype distribution. According to **Wei et al.** [11], the genetic variant ZFHX3 (rs2106261) was associated with an increased risk of AF and consequently CI in Asian and Caucasian samples, with Asian individuals showing a stronger association, but no association was found in African samples.

Finally, the limitations of our study were the small sample size and small number of SNPs. Therefore, it is necessary to conduct further studies with larger sample sizes to assess the possible effect of SNPs on the severity of COVID-19.

## Conclusion

CDKN2B-AS1 (rs1333049) gene polymorphism may play a role in determining the degree of COVID-19 severity. Further studies on its effect on cyclins and CDKs [not measured in our study] may shed light on new treatment options for COVID-19.

### Electronic supplementary material

Below is the link to the electronic supplementary material.

Supplementary Material 1

## Data Availability

The datasets generated during the current study are available on NCBI under the dbSNP repository, Submission ID: SUB12958873 [https://submit.ncbi.nlm.nih.gov/subs/variation_file/SUB12958873/].

## Abbreviations

COVID-19: Coronavirus disease 2019
MI: Myocardial infarction
AF: Atrial fibrillation
CI: Cerebral infarction
CDKN2B-AS1: Cyclin-dependent kinase inhibitor 2B antisense RNA 1
ZFHX3: Zinc finger homeobox 3
SNPs: Single nucleotide polymorphisms
CDKs: Cyclin-dependent kinases
CoV: Coronavirus
SARS-CoV-1: Severe acute respiratory syndrome-related coronavirus-1
MERS-CoV: Middle East respiratory syndrome coronavirus
SARS-CoV-2: severe acute respiratory syndrome-related coronavirus-2
SARS: Special administrative regions
IMCU: Intermediate care unit
ICU: Intensive care unit
COPD: Chronic obstructive pulmonary disease
DM: Diabetes mellitus
GWASs: Genome-wide association studies
CAD: Coronary artery disease
CDKN2B: Cyclin-dependent kinase inhibitor 2B
HTN: Hypertension
CAK: CDK activating kinase
SMCs: Smooth muscle cells
ARDS: Acute respiratory distress syndrome
NETs: Neutrophil extracellular traps
CBC: Complete blood count
INR: International normalized ratio
PC%: Prothrombin concentration percentage
IQR: Interquartile range

## References

[1] Jacob CO (2020). On the genetics and immunopathogenesis of COVID-19. Clin Immunol.

[2] Zhou P, Yang XL, Wang XG, Hu B, Zhang L, Zhang W, Si HR, Zhu Y, Li B, Huang CL, Chen HD, Chen J, Luo Y, Guo H, Jiang RD, Jiang RD, Liu MQ, Chen Y, Shen XR, Wang X, Zheng XS, Zhao K, Chen QJ, Deng F, Liu LL, Yan B, Zhan FX, Wang YY, Xiao GF, Shi ZL (2020). A pneumonia outbreak associated with a new coronavirus of probable bat origin. Nature.

[3] Synowiec A, Szczepański A, Barreto-Duran E, Lie LK, Pyrc K (2021). Severe Acute Respiratory Syndrome Coronavirus 2 (SARS-CoV-2): a systemic infection. Clin Microbiol Rev.

[4] Dhama K, Patel SK, Sharun K, Pathak M, Tiwari R, Yatoo MI, Malik YS, Sah R, Rabaan AA, Panwar PK, Singh KP, Michalak I, Chaicumpa W, Martinez-Pulgarin DF, Bonilla-Aldana DK, Rodriguez-Morales AJ (2020). SARS-CoV-2 jumping the species barrier: zoonotic lessons from SARS, MERS and recent advances to combat this pandemic virus. Travel Med Infect Dis.

[5] Guan W, Ni Z, Hu Y, Liang WH, Ou CQ, He JX, Liu L, Shan H, Lei CI, Hui DSC, Du B, Li LJ, Zeng G, Yuen KY, Chen RC, Tang CI, Wang T, Chen PY, Xiang J, Li SY, Wang JI, Liang ZJ, Peng YX, Wei L, Liu Y, Hu YH, Peng P, Wang JM, Liu JY, Chen Z, Li G, Zheng ZJ, Qiu SQ, Luo J, Ye CJ, Zhu SY, Zhong NS (2020). Clinical characteristics of coronavirus disease 2019 in China. N Engl J Med.

[6] Meister T, Pisarev H, Kolde R, Kalda R, Suija K, Milani L, Karo-Astover L, Piirsoo M, Uusküla A (2022). Clinical characteristics and risk factors for COVID-19 infection and disease severity: a nationwide observational study in Estonia. PLoS ONE.

[7] Petrilli CM, Jones SA, Yang J, Rajagopalan H, O’Donnell L, Chernyak Y, Tobin KA, Cerfolio RJ, Francois F, Horwitz LI (2020). Factors associated with hospital admission and critical illness among 5279 people with coronavirus disease 2019 in New York City: prospective cohort study. BMJ.

[8] Suleyman G, Fadel RA, Malette KM, Hammond C, Abdulla H, Entz A, Demertzis Z, Hanna Z, Failla A, Dagher C, Chaudhry Z (2020). Clinical characteristics and morbidity associated with coronavirus disease 2019 in a series of patients in metropolitan Detroit. JAMA Netw open.

[9] Abobaker A, Nagib T, Alsoufi A (2021). The impact of certain genetic variants (single nucleotide polymorphisms) on incidence and severity of COVID-19. J Gene Med.

[10] Yahia M, El Abd A, Hamouda MA, Abd El EM (2021). Association of CDKN2B-AS1 gene polymorphism with Acute myocardial infarction. J Cardiovasc Disease Res.

[11] Wei Y, Wang L, Lin C, Xie Y, Bao Y, Luo Q, Zhang N (2021). Association between the rs2106261 polymorphism in the zinc finger homeobox 3 gene and risk of atrial fibrillation: evidence from a PRISMA-compliant meta-analysis. Medicine.

[12] Yari A, Saleh-Gohari N, Mirzaee M, Hashemi F, Saeidi K (2021). A study of Associations between rs9349379 (PHACTR1), rs2891168 (CDKN2B-AS), rs11838776 (COL4A2) and rs4880 (SOD2) polymorphic Variants and Coronary Artery Disease in Iranian Population. Biochem Genet.

[13] Kaur N, Singh J, Reddy S (2020). ANRIL rs1333049 C/G polymorphism and coronary artery disease in a north indian population - gender and age specific associations. Genet Mol Biol.

[14] Ou M, Li X, Zhao S, Cui S, Tu J (2020). Long non-coding RNA CDKN2B-AS1 contributes to atherosclerotic plaque formation by forming RNA-DNA triplex in the CDKN2B promoter. EBioMedicine.

[15] Youn BJ, Cheong HS, Namgoong S, Kim LH, Baek IK, Kim JH, Yoon SJ, Kim EH, Kim SH, Chang JH, Kim SH. Asian-specific 3’UTR variant in CDKN2B associated with risk of pituitary adenoma. Mol Biol Rep. (2022):1–8. 10.1007/s11033-022-07796-1 36097105

[16] Kojima Y, Ye J, Nanda V, Wang Y, Flores AM, Jarr KU, Tsantilas P, Guo L, Finn AV, Virmani R, Leeper NJ (2020). Knockout of the murine ortholog to the human 9p21 coronary artery disease locus lGrinshpun eads to smooth muscle cell proliferation, vascular calcification, and advanced atherosclerosis. Circulation.

[17] Gupta R, Mlcochova P (2022). Cell cycle independent role of cyclin D3 in host restriction of SARS-CoV-2 infection. bioRxiv.

[18] Klinger J, Ravarani C, Bannard C, Lamparter M, Schwinges A, Cope J, Baukmann H, Schmidt M. Critically ill COVID-19 status associated trait genetics reveals CDK6 inhibitors as potential treatment. (2021).

[19] Grinshpun A, Merlet I, Fruchtman H, Nachman D. A protracted course of COVID19 infection in a metastatic breast Cancer Patient during CDK4/6 inhibitor therapy. Front Oncol. (2020);10:1085. 10.3389/fonc.2020.01085 PMC7295974 32582559

[20] Sule WF, Oluwayelu DO (2020). Real-time RT-PCR for COVID-19 diagnosis: challenges and prospects. Pan Afr Med J.

[21] Salunke AA, Warikoo V, Kumar Pathak S, Nandy K, Mujawar J, Mendhe H, Shah A, Kottakota V, Menon V, Pandya S (2020). A proposed ABCD scoring system for better triage of patients with COVID-19: use of clinical features and radiopathological findings. Diabetes Metab Syndr.

[22] Tomomori S, Nakano Y, Ochi H, Onohara Y, Sairaku A, Tokuyama T, Motoda C, Matsumura H, Amioka M, Hironobe N, Ookubo Y (2018). Maintenance of low inflammation level by the ZFHX3 SNP rs2106261 minor allele contributes to reduced atrial fibrillation recurrence after pulmonary vein isolation. PLoS ONE.

[23] Hu J, Li C, Wang S, Li T, Zhang H (2021). Genetic variants are identified to increase risk of COVID-19 related mortality from UK Biobank data. Hum Genomics.

[24] Palladino M (2021). Complete blood count alterations in COVID-19 patients: a narrative review. Biochem Med (Zagreb).

[25] Soraya GV, Ulhaq ZS (2020). Crucial laboratory parameters in COVID-19 diagnosis and prognosis: an updated meta-analysis. Med Clin (Barc).

[26] Zhu B, Feng X, Jiang C, Mi S, Yang L, Zhao Z, Zhang Y, Zhang L (2021). Correlation between white blood cell count at admission and mortality in COVID-19 patients: a retrospective study. BMC Infect Dis.

[27] Lippi G, Plebani M, Henry BM (2020). Thrombocytopenia is associated with severe coronavirus disease 2019 (COVID-19) infections: a meta-analysis. Clin Chim Acta.

[28] Zaid Y, Puhm F, Allaeys I, Naya A, Oudghiri M, Khalki L, Limami Y, Zaid N, Sadki K, El Ben R, Mahir W (2020). Platelets can associate with SARS-Cov-2 RNA and are hyperactivated in COVID-19. Circul Res.

[29] Wang L, Duan Y, Zhang W, Liang J, Xu J, Zhang Y, Wu C, Xu Y, Li H. Epidemiologic and clinical characteristics of 26 cases of COVID-19 arising from patient-to-patient transmission in Liaocheng, China. Clinical epidemiology. (2020);12:387–91. 10.2147/CLEP.S249903 PMC7154005 32308494

[30] Huang Y, Tu M, Wang S, Chen S, Zhou W, Chen D, Zhou L, Wang M, Zhao Y, Zeng W, Huang Q (2020). Clinical characteristics of laboratory confirmed positive cases of SARS-CoV-2 infection in Wuhan, China: a retrospective single center analysis. Travel Med Infect Dis.

[31] Pazgan-Simon M, Serafińska S, Kukla M, Kucharska M, Zuwała-Jagiełło J, Buczyńska I, Zielińska K, Simon K (2022). Liver injury in patients with COVID-19 without underlying liver disease. J Clin Med.

[32] Liu YM, Xie J, Chen MM, Zhang X, Cheng X, Li H, Zhou F, Qin JJ, Lei F, Chen Z, Lin L (2021). Kidney function indicators predict adverse outcomes of COVID-19. Med.

[33] Amin A, Moon R, Agiro A, Rosenthal N, Brown H, Legg R, Pottorf W (2022). In-hospital mortality, length of stay, and hospitalization cost of COVID-19 patients with and without hyperkalemia. Am J Med Sci.

[34] Islam MK, Hasan P, Sharif MM, Khan TD, Ratul RH, Hossain FS, Molla MMA (2022). Hyponatremia in COVID-19 patients: experience from Bangladesh. Health Sci Rep.

[35] Mahat RK, Rathore V, Singh N, Singh N, Singh SK, Shah RK, Garg C (2021). Lipid profile as an indicator of COVID-19 severity: a systematic review and meta-analysis. Clin Nutr ESPEN.

[36] Zinellu A, Paliogiannis P, Fois AG, Solidoro P, Carru C, Mangoni AA (2021). Cholesterol and triglyceride concentrations, COVID-19 severity, and mortality: a systematic review and Meta-analysis with Meta-regression. Front Public Health.

[37] Wei X, Zeng W, Su J, Wan H, Yu X, Cao X, Tan W, Wang H (2020). Hypolipidemia is associated with the severity of COVID-19. J Clin Lipidol.

[38] Zinellu A, Paliogiannis P, Carru C, Mangoni AA (2021). INR and COVID-19 severity and mortality: a systematic review with meta-analysis and meta-regression. Adv Med Sci.

[39] Liu SL, Wang SY, Sun YF, Jia QY, Yang CL, Cai PJ, Li JY, Wang L, Chen Y. Expressions of SAA, CRP, and FERR in different severities of COVID-19. European review for Medical and Pharmacological Sciences. (2020);24(21):11386–94. 10.26355/eurrev_202011_23631 33215460

