# Supplementary material for: Association between cyclin-dependent kinase inhibitor 2B antisense RNA 1 and zinc finger homeobox 3 gene polymorphisms and COVID-19 severity
Source: BMC Infect Dis. 2023 Aug 31;23:568. doi: 10.1186/s12879-023-08564-7 (PMC10472581; doi:10.1186/s12879-023-08564-7)
Supplement: Supplementary file 1 — Supplementary Material 1 [file 12879_2023_8564_MOESM1_ESM.docx]

**Table S 1: Associated comorbidities and prognosis of the COVID-19 cases:**

|  |  | **Severe COVID-19 cases (n= 90)** | | **Moderate COVID-19 cases (n= 90)** | | **χ^2^** | **p** |
| --- | --- | --- | --- | --- | --- | --- | --- |
|  |  | **No.** | **%** | **No.** | **%** |  |  |
| **Associated comorbidities** | **-Heart disease** |  |  |  |  |  |  |
|  | No | 58 | 64.4 | 84 | 93.3 | 22.55^*^ | <0.001^*^ |
|  | Yes | 32 | 11.1 | 6 | 2.2 |  |  |
|  | **-DM** | 52 | 57.8 | 28 | 31.1 | 12.96^*^ | <0.001^*^ |
|  | **-HTN** | 66 | 73.3 | 26 | 28.9 | 35.573^*^ | <0.001^*^ |
|  | **Old stroke** | 8 | 8.9 | 4 | 4.4 | 1.429 | ^FE^p=0.371 |
|  | **COPD** | 16 | 17.8 | 8 | 8.9 | 3.077 | 0.079 |
| **Prognosis** | |  |  |  |  |  |  |
| Improved | | 28 | 31.1 | 54 | 60.0 | 15.142^*^ | <0.001^*^ |
| Death | | 62 | 68.9 | 36 | 40.0 |  |  |

χ^2^: **Chi square test**  FE: **Fisher Exact**

p: p value for comparing **the two studied groups**

*: Statistically significant at p ≤ 0.05

**ICM:** Ischemic cardiomyopathy **IHD:** [Ischemic Heart Disease](file://E:\\corona\\المعمل المركزى\\corona paper.docx" \l ":~:text=What is ischemic heart disease,disease and coronary heart disease.)

**IHD &IDCM:** Ischemic Heart Disease & idiopathic dilated cardiomyopathy

**DM:** diabetes mellitus **HTN:** hypertension

**COPD:** [Chronic obstructive pulmonary disease](file://E:\\corona\\المعمل المركزى\\corona paper.docx" \l ":~:text=Chronic obstructive pulmonary disease, or,Americans who have this disease.) **[ARDS:](file://E:\\corona\\المعمل المركزى\\corona paper.docx" \l ":~:text=Chronic obstructive pulmonary disease, or,Americans who have this disease.)** [Acute respiratory distress syndrome](file://E:\\corona\\المعمل المركزى\\corona paper.docx" \l ":~:text=Chronic obstructive pulmonary disease, or,Americans who have this disease.)

**AKI:** Acute kidney injury  **AF:** Atrial fibrillation

**DKA:** Diabetic ketoacidosis **DCL:** Disturbed conscious level

**CORAD:** Coronavirus Disease 2019 (COVID-19) Reporting and Data System

**Table S 2: Relation between ZFHX3 (rs2106261) gene polymorphism and laboratory data and prognosis in moderate COVID-19 cases (n= 90):**

|  | **Reference range** | **rs2106261** | | **Test of sig.** | **p** |
| --- | --- | --- | --- | --- | --- |
|  |  | **C/C (n= 66)** | **C/T (n= 24)** |  |  |
| **WBCs** | 4.00-11/(mcL) |  |  |  |  |
| Median |  | 10.0 | 13.40 | U=556.0^*^ | 0.031^*^ |
| **Platelet** | 150-400/(mcL) |  |  |  |  |
| Median |  | 231.0 | 214.50 | U=670.0 | 0.265 |
| **HB (g/dl)** | Male: 14-18 g/dL |  |  |  |  |
| Mean ± SD. | Female: 12-16 g/dL | 11.58 ± 2.13 | 11.52 ± 2.18 | t=0.122 | 0.904 |
| **SGOT (U/L)** | 8-45 (U/L) |  |  |  |  |
| Median |  | 38.0 | 35.0 | U=652.0 | 0.201 |
| **SGPT (U/L)** | 7-56 (U/L) |  |  |  |  |
| Median |  | 30.0 | 33.50 | U=654.0 | 0.208 |
| **BUN (mg/dL)** | 6-24 mg/dL |  |  |  |  |
| Median |  | 28.0 | 25.50 | U=724.0 | 0.535 |
| **Creatinine (mg/dL)** | Male: .6-1.2 mg/dL |  |  |  |  |
| Median | Female: .5 to 1.1 mg/dL | 0.80 | 1.05 | U=508.0^*^ | 0.009^*^ |
| **Na^+^ (mEq/L)** | 135-145 (mEq/L) |  |  |  |  |
| Mean ± SD. |  | 139.0 ± 5.92 | 135.58 ± 4.46 | t=2.570^*^ | 0.012^*^ |
| **K^+^ (mEq/L)** | 3.5-5.3 (mEq/L |  |  |  |  |
| Mean ± SD. |  | 4.26 ± 0.73 | 4.16 ± 0.65 | t=0.581 | 0.563 |
| **TG (mg/dL)** | Male: 40-160 mg/dL |  |  |  |  |
| Median | Female: 35-135 mg/dL | 132.0 | 115.0 | U=658.0 | 0.221 |
| **Cholesterol (mg/dL)** | 125-200 mg/dL |  |  |  |  |
| Median |  | 111.0 | 101.50 | U=580.0 | 0.052 |
| **HDL (mg/dL)** | Male: 35-55 mg/dL |  |  |  |  |
| Median | Female: 45-65 mg/dL | 35.0 | 30.0 | U=658.0 | 0.220 |
| **LDL (mg/dL)** | < 130 mg/dL |  |  |  |  |
| Median |  | 51.0 | 45.20 | U=640.0 | 0.165 |
| **PC%** |  |  |  |  |  |
| Mean ± SD. |  | 78.30 ± 17.85 | 80.08 ± 13.82 | t=0.443 | 0.659 |
| **INR** | 0.9–1.1 |  |  |  |  |
| Median |  | 1.18 | 1.13 | U=770.50 | 0.841 |
| **CRP (mg/L)** | < 3 mg/L |  |  |  |  |
| Median |  | 48.0 | 49.0 | U=754.0 | 0.728 |
| **Ferritin (µg/L)** | Male: 24-336 (µg/L) |  |  |  |  |
| Median | Female: 11-307 (µg/L) | 460.0 | 335.0 | U=750.0 | 0.701 |
| **D-dimer (µg/mL)** | 0-0.50 (µg/mL) |  |  |  |  |
| Median |  | 0.90 | 0.81 | U=584.0 | 0.059 |
| **Prognosis** |  |  |  |  |  |
| Improved |  | 42 (63.6%) | 12 (50%) | χ^2^=1.363 | 0.243 |
| Death |  | 24 (36.45%) | 12 (50%) |  |  |

SD: **Standard deviation** t: **Student’s t test**

U: **Mann‒Whitney test** χ2: **Chi square test**

p: p value for comparison between the studied categories

*: Statistically significant at p ≤ 0.05

**WBCs:** White blood cells  **HB:** Hemoglobin

**SGOT:** Serum glutamic-oxaloacetic transaminase  **SGPT:** Serum glutamic-pyruvic transaminase

**BUN:** [Blood urea nitrogen](https://www.mayoclinic.org/tests-procedures/blood-urea-nitrogen/about/pac-20384821)  **[TG:](https://www.mayoclinic.org/tests-procedures/blood-urea-nitrogen/about/pac-20384821)** [Triglyceride](https://www.mayoclinic.org/tests-procedures/blood-urea-nitrogen/about/pac-20384821)

**HDL:** High-density lipoprotein **LDL:** Low-density lipoprotein

**PC%:** prothrombin concentration percentage **INR:** International normalized ratio

**CRP:** c-reactive protein

**Table S 3: Relationship between prognosis and demographic and laboratory data in severe COVID-19 cases (n= 90):**

|  | **Reference range** | **Prognosis** | | **Test of sig.** | **p** |
| --- | --- | --- | --- | --- | --- |
|  |  | **Improved (n= 28)** | **Death (n= 62)** |  |  |
| **Sex** |  |  |  |  |  |
| Male |  | 14 (50%) | 20 (32.3%) | χ^2^=2.583 | 0.108 |
| Female |  | 14 (50%) | 42 (67.7%) |  |  |
| **Age (years)** |  |  |  |  |  |
| Mean ± SD. |  | 64.71 ± 7.23 | 65.81 ± 6.94 | t=0.672 | 0.505 |
| **WBCs** | 4.00-11/(mcL) |  |  |  |  |
| Median |  | 11.40 | 9.70 | U=716.0 | 0.185 |
| **Platelet** | 150-400/(mcL) |  |  |  |  |
| Median |  | 192.0 | 209.0 | U=738 | 0.257 |
| **HB (g/dl)** | Male: 14-18 g/dL |  |  |  |  |
| Mean ± SD. | Female: 12-16 g/dL | 10.78 ± 1.86 | 10.58 ± 1.89 | t=0.454 | 0.651 |
| **SGOT (U/L)** | 8-45 (U/L) |  |  |  |  |
| Median |  | 37.0 | 39.0 | U=782.0 | 0.453 |
| **SGPT (U/L)** | 7-56 (U/L) |  |  |  |  |
| Median |  | 30.50 | 26.0 | U=762.0 | 0.355 |
| **BUN (mg/dL)** | 6-24 mg/dL |  |  |  |  |
| Median |  | 32.0 | 49.0 | U=746.0 | 0.287 |
| **Creatinine (mg/dL)** | Male: .6-1.2 mg/dL |  |  |  |  |
| Median | Female: .5 to 1.1 mg/dL | 1.25 | 1.30 | U=856.0 | 0.917 |
| **Na^+^ (mEq/L)** | 135-145 (mEq/L) |  |  |  |  |
| Mean ± SD. |  | 139.29 ± 3.86 | 140.59 ± 6.01 | t=1.055 | 0.294 |
| **K^+^ (mEq/L)** | 3.5-5.3 (mEq/L |  |  |  |  |
| Mean ± SD. |  | 4.56 ± 0.81 | 4.40 ± 0.64 | t=1.056 | 0.294 |
| **TG (mg/dL)** | Male: 40-160 mg/dL |  |  |  |  |
| Median | Female: 35-135 mg/dL | 134.0 | 114.0 | U=494.0^*^ | 0.001^*^ |
| **Cholesterol (mg/dL)** | 125-200 mg/dL |  |  |  |  |
| Median |  | 128.0 | 107.50 | U=526.0^*^ | 0.003^*^ |
| **HDL (mg/dL)** | Male: 35-55 mg/dL |  |  |  |  |
| Median | Female: 45-65 mg/dL | 35.50 | 35.0 | U=684.0 | 0.108 |
| **LDL (mg/dL)** | < 130 mg/dL |  |  |  |  |
| Median |  | 59.95 | 42.34 | U=554.0^*^ | 0.006^*^ |
| **PC%** |  |  |  |  |  |
| Mean ± SD. |  | 74.82 ± 20.51 | 72.60 ± 16.72 | t=0.543 | 0.589 |
| **INR** | 0.9–1.1 |  |  |  |  |
| Median |  | 1.20 | 1.20 | U=816.0 | 0.650 |
| **CRP (mg/L)** | < 3 mg/L |  |  |  |  |
| Median |  | 48.0 | 70.0 | U=810.0 | 0.612 |
| **Ferritin (µg/L)** | Male: 24-336 (µg/L) |  |  |  |  |
| Median | Female: 11-307 (µg/L) | 422.50 | 454.0 | U=764.0 | 0.365 |
| **D-dimer (µg/mL)** | 0-0.50 (µg/mL) |  |  |  |  |
| Median |  | 1.06 | 0.94 | U=818.0 | 0.663 |

χ2: **Chi square test** SD: **Standard deviation**

t: **Student’s t test** U: **Mann‒Whitney test**

p: p value for comparison between the studied categories

*: Statistically significant at p ≤ 0.05

**WBCs:** White blood cells  **HB:** Hemoglobin

**SGOT:** Serum glutamic-oxaloacetic transaminase  **SGPT:** Serum glutamic-pyruvic transaminase

**BUN:** [Blood urea nitrogen](https://www.mayoclinic.org/tests-procedures/blood-urea-nitrogen/about/pac-20384821)  **[TG:](https://www.mayoclinic.org/tests-procedures/blood-urea-nitrogen/about/pac-20384821)** [Triglyceride](https://www.mayoclinic.org/tests-procedures/blood-urea-nitrogen/about/pac-20384821)

**HDL:** High-density lipoprotein **LDL:** Low-density lipoprotein

**PC%:** prothrombin concentration percentage **INR:** International normalized ratio

**CRP:** c-reactive protein

**Table S 4: Relationship between prognosis and demographic and laboratory data in moderate COVID-19 cases (n= 90):**

|  | **Reference range** | **Prognosis** | | **Test of sig.** | **p** |
| --- | --- | --- | --- | --- | --- |
|  |  | **Improved (n= 54)** | **Death (n= 36)** |  |  |
| **Sex** |  |  |  |  |  |
| Male |  | 26 (48.1%) | 22 (61.1%) | χ^2^=1.4583 | 0.227 |
| Female |  | 28 (51.9%) | 14 (38.9%) |  |  |
| **Age (years)** |  |  |  |  |  |
| Mean ± SD. |  | 53.07 ± 16.93 | 65.83 ± 15.23 | t=3.721**^*^** | <0.001**^*^** |
| **WBCs** | 4.00-11/(mcL) |  |  |  |  |
| Median |  | 8.90 | 13.40 | U=514.0^*^ | <0.001^*^ |
| **Platelet** | 150-400/(mcL) |  |  |  |  |
| Median |  | 255.0 | 215.0 | U=810.0 | 0.182 |
| **HB (g/dl)** | Male: 14-18 g/dL |  |  |  |  |
| Mean ± SD. | Female: 12-16 g/dL | 11.31 ± 2.08 | 11.93 ± 2.18 | t=1.355 | 0.179 |
| **SGOT (U/L)** | 8-45 (U/L) |  |  |  |  |
| Median |  | 33.0 | 40.0 | U=892.0 | 0.510 |
| **SGPT (U/L)** | 7-56 (U/L) |  |  |  |  |
| Median |  | 30.0 | 37.50 | U=856.0 | 0.339 |
| **BUN (mg/dL)** | 6-24 mg/dL |  |  |  |  |
| Median |  | 23.0 | 30.50 | U=676.0^*^ | 0.014^*^ |
| **Creatinine (mg/dL)** | Male: .6-1.2 mg/dL |  |  |  |  |
| Median | Female: .5 to 1.1 mg/dL | 0.80 | 1.24 | U=668.0^*^ | 0.012^*^ |
| **Na^+^ (mEq/L)** | 135-145 (mEq/L) |  |  |  |  |
| Mean ± SD. |  | 138.0 ± 4.69 | 138.22 ± 7.12 | t=0.179 | 0.859 |
| **K^+^ (mEq/L)** | 3.5-5.3 (mEq/L |  |  |  |  |
| Mean ± SD. |  | 4.31 ± 0.70 | 4.12 ± 0.71 | t=1.298 | 0.198 |
| **TG (mg/dL)** | Male: 40-160 mg/dL |  |  |  |  |
| Median | Female: 35-135 mg/dL | 138.0 | 105.0 | U=608.0^*^ | 0.003^*^ |
| **Cholesterol (mg/dL)** | 125-200 mg/dL |  |  |  |  |
| Median |  | 115.0 | 102.0 | U=726.0^*^ | 0.042^*^ |
| **HDL (mg/dL)** | Male: 35-55 mg/dL |  |  |  |  |
| Median | Female: 45-65 mg/dL | 35.0 | 33.0 | U=934.0 | 0.754 |
| **LDL (mg/dL)** | < 130 mg/dL |  |  |  |  |
| Median |  | 50.50 | 43.78 | U=794.0 | 0.143 |
| **PC%** |  |  |  |  |  |
| Mean ± SD. |  | 78.29 ± 18.33 | 79.50 ± 14.46 | t=0.332 | 0.741 |
| **INR** | 0.9–1.1 |  |  |  |  |
| Median |  | 1.18 | 1.15 | U=946.0 | 0.830 |
| **CRP (mg/L)** | < 3 mg/L |  |  |  |  |
| Median |  | 48.0 | 50.0 | U=852.0 | 0.322 |
| **Ferritin (µg/L)** | Male: 24-336 (µg/L) |  |  |  |  |
| Median | Female: 11-307 (µg/L) | 400.0 | 459.50 | U=778.0 | 0.110 |
| **D-dimer (µg/mL)** | 0-0.50 (µg/mL) |  |  |  |  |
| Median |  | 0.90 | 0.8 | U=780.0 | 0.113 |

χ2: **Chi square test** SD: **Standard deviation**

t: **Student’s t test** U: **Mann‒Whitney test**

p: p value for comparison between the studied categories

*: Statistically significant at p ≤ 0.05

**WBCs:** White blood cells  **HB:** Hemoglobin

**SGOT:** Serum glutamic-oxaloacetic transaminase  **SGPT:** Serum glutamic-pyruvic transaminase

**BUN:** [Blood urea nitrogen](https://www.mayoclinic.org/tests-procedures/blood-urea-nitrogen/about/pac-20384821)  **[TG:](https://www.mayoclinic.org/tests-procedures/blood-urea-nitrogen/about/pac-20384821)** [Triglyceride](https://www.mayoclinic.org/tests-procedures/blood-urea-nitrogen/about/pac-20384821)

**HDL:** High-density lipoprotein **LDL:** Low-density lipoprotein

**PC%:** prothrombin concentration percentage **INR:** International normalized ratio

**CRP:** c-reactive protein

**Table S5: Pairwise linkage disequilibrium of gene polymorphisms:**

|  | **D** | **D'** | **R** | **R^2^** | **χ^2^** | **p** |
| --- | --- | --- | --- | --- | --- | --- |
| **Group 1a** | 0.059 | 0.639 | 0.301 | 0.091 | 8.187 | 0.004^*^ |
| **Group 1b** | 0.040 | 0.643 | 0.236 | 0.056 | 5.007 | 0.025^*^ |
| **Group 2** | 0.600 | 0.107 | 0.058 | 0.003 | 0.600 | 0.438 |

χ^2^: **Chi square test**

D: Linkage disequilibrium

D': Standardization disequilibrium

R: coefficient of regression

R^2^: Coefficient of determination

There was a significant increase in the frequency of MI, HTN and DM (P<0.001) in the severe COVID-19 patients compared with the moderate COVID-19 patients, and there was no significant difference regarding other associated comorbidities (old stroke and COPD). Regarding prognosis, there was a significant increase in death among severe COVID-19 patients compared with moderate COVID-19 patients **(Table S 1).**

Regarding the relation of ZFHX3 (rs2106261) genotype distribution in moderate COVID-19 patients, there was a significant association of higher WBC count (P=0.031), higher creatinine (P=0.009) and lower Na+ level (P=0.012) in the C/T genotype compared to the C/C genotype, while there was no significant association regarding other parameters **(Table S2).**

Regarding the relationship of prognosis in severe COVID-19 patients, there was a significant association of lower TG (P=0.001), lower total cholesterol (p=0.003) and lower LDL (P=0.006) in dead COVID-19 patients, while there was no significant association regarding other parameters **(Table S3).**

Regarding the relationship of prognosis in moderate COVID-19 patients, there was a significant association of older age (P<0.001), higher WBC count (P<0.001), higher BUN (P=0.014), higher creatinine (P=0.012), lower TG, (P=0.003) and lower total cholesterol (P=0.042) in deceased COVID-19 patients, while there was no significant association regarding other parameters **(Table S4).**

The linkage disequilibrium (LD) test (D prime (D’) approximately 0.64) between CDKN2B-AS1 (rs1333049) and ZFHX3 (rs2106261) showed that they are strongly linked to each other in severe and moderate COVID-19 patients. However, they were not linked in the control group **(Table S5).**
